# The Wastage from Disease in the American Expeditionary Forces in the Year June 1917 to May 1918

**Published:** 1918-09

**Authors:** Haven Emerson


**The Wastage from Disease in the American Expeditionary Forces in the Year June 1917 to May 1918.** By Major Haven Emerson, M. R. C.

The following is a detailed summary of Major Emerson's Report:

If there had been no loss of service on account of sickness in the A. E. F. in the year ending May 31st, 1918, 62,714,000 days of service would have been given. As a matter of fact, 1,481,000 days of service were lost to the A. E. F., as a result of sickness, that is, 2.377 °/₀ of total possible days of service. There have been, of course, losses from battle casualties and from accidents and injuries suffered in the course of industries and transportation carried out by members of the A. E. F. These losses are not included in this report. Of the total days lost on account of sickness, 1,403,130 were lost in hospital and 77,870 in quarters. The days lost were due to 13[illegible],075 cases of sickness resulting in 923 deaths (.07 °/₀); 110,687 returned to duty (84.36 °/₀); otherwise disposed of by discharge, return to the United States, or in other ways (15.55 °/₀).

The following list of diseases or disease groups represents 77.11 °/₀ of the total days lost from sickness, that is 1,142,288 days. The order in which the diseases appear in the list is the order of their importance measured by the numbers of days of sick wastage caused by these diseases in the past year.

1. Mumps.
2. Bronchitis.
3. Venereal Disease.
4. Laryngitis, Pharyngitis, Tonsilitis.
5. Pneumonia-Lobar.
6. Measles.
7. Tuberculosis (all kinds).
8. Arthritis, Synovitis (acute and chronic).
9. Rheumatic Fever (acute).
10. Otitis Media.
11. Hernia.
12. Enteritis, Colitis, Gastritis.
13. Broncho-Pneumonia.
14. Appendicitis (purulant and catarrhal).
15. Scarlatina.
16. Not yet diagnosed.
17. Pleurisy acute fibrinous.
18. Skin Diseases (other).
19. Hemorrhoids.
20. Flat Foot.
21. Valvular Disease of the heart.
22. Diphtheria and Carriers.
23. Heart Disease (other).
24. Mental Disease.
25. Scabies.
26. Furunculosis and Pyodermias.
27. Meningitis and Carriers.
28. Abscess of Skin.
29. Sinusitis.
30. Abscess, peritonsillar.
31. Phimosis.
32. Lymphadenitis.
33. Conjunctivitis.
34. Trench Foot and Chilblain.
35. Mastoiditis.
36. Fever of Unknown Origin (Trench Fever).
37. Orchitis.
38. Hypertrophied Tonsils.
39. German Measles.
40. Nephritis Acute and Chronic.
41. Dysentery.
42. Stomatitis and Gingivitis.

Of the total number of days lost from sickness, 488,033 days or 32.41 % were lost from the following preventable diseases :

Diphtheria and Carriers.

Dysentery.

German Measles.

Measles.

Meningitis and Carriers.

Mumps.

Fever of Unknown Origin (Trench Fever).

Venereal diseases (Chancroid, Gonorrhea and Syphilis).

Another 284,458 days or 19.20 % were lost from other diseases resulting from preventable and unfavorable conditions of personal environment, such conditions as are not always controllable in Military operations; namely, overcrowding in quarters, exposure to cold and wet and fatigue. The " maturity " or " seasoning " of troops will diminish these diseases, but that is generally another way of expressing the steady improvement in the personal hygiene of troops as the line officers are educated in the necessity of ceaseless and painstaking vigilance and forethought. The diseases referred to are :

Pneumonia Lobar.

" broncho.

Bronchitis acute.

Pleurisy acute.

Acute Rhinitis.

" Pharyngitis.

" Laryngitis.

" Tonsilitis.

Secondary in many instances to one or more of the above group of affections of the respiratory tract are :

Sinusitis.

Otitis Media.

Mastoiditis.

which add a total of 36,390 days lost or 2.45 % of total days lost.

During the year ending May 31st, 1918, there have been 34,892 cases of the following communicable and preventable diseases in the A. E. F., 1,214 among Officers and 32,668 among enlisted men.

Pneumonia.

Meningitis.

Measles.

Mumps.

Diphtheria.

Influenza.

This is 34.30 % of all cases other than those due to injury and in action.

These six communicable diseases need no intermediate host or other means of transmission than the direct conveyance from the sick or from early and undeclared cases to the well, of the discharges from the nose and mouth, that is, the moist spray and the spit.

In the case of diphtheria, mumps, and measles, a practical immunity is conferred by an attack of the disease, but this is not the case with the other four diseases mentioned, and it is furthermore recognized that an attack of influenza or pneumonia renders the subject more and not less susceptible to subsequent attacks.

We may expect a decidedly lower incidence of measles and mumps next year in the organizations which experienced epidemics of these diseases in the United States or France during the past year; but the recruiting and arrival in France of much larger numbers of men, transported under at least as unfavourable conditions of crowding on shipboard as were those a year ago, will doubtless reestablish widespread epidemics of these diseases unless radical improvements are made in the matter of barrack crowding, and in facilities for holding of exposed or contact cases for observation apart from the rest of a command.

There is no reason to expect any material reduction of the other diseases, i. e., diphtheria, influenza, meningitis, pneumonia, simply as a result of a longer experience by our troops of the climatic conditions in France, for scarcely 10 % of the expected strength for the winter of 1918-19 was in France in the winter of 1917-18.

It is to be noted particularly that although the officers represent about 6 % of the total strength, they represent only 3.4 % of the cases of the diseases under consideration and only 12 % of the pneumonia, a disease which may properly be said to occur in direct proportion to the degree of overcrowding in living quarters.

By correspondence with the D. D. M. S. Sanitation of the B. E. F. the incidence and death rate for pneumonia in the B. E. F. has been obtained and is here compared with corresponding rates in the A. E. F. for the months of January, February and March 1918, the months of the annual high pneumonia incidence in temperate climates.

*American Expeditionary Forces.*

| | No. of cases of Pneumonia per 100,000 per month. | No. of deaths from Pneumonia per 100,000 per month. | No. of deaths from Pneumonia per 100 cases of Pneumonia. |
|---|---|---|---|
| Jan. . . . . . . . . | 258.73 | 85.71 | 33.13 |
| Feb. . . . . . . . . | 96.89 | 32.89 | 33.95 |
| March. . . . . . . . | 175.81 | 48.74 | 27.72 |

*British Expeditionary Force.*

| | | | |
|---|---|---|---|
| Jan. . . . . . . . . | 17.11 | 2.43 | 14.19 |
| Feb. . . . . . . . . | 5.65 | .31 | 5.66 |
| March . . . . . . | 10.11 | .47 | 4.69 |

An additional evidence of the seriousness of the situation with regard to pneumonia in the A. E. F. is to be seen in a comparison with case and death rates in the United States Army in the period 1905-14 and for the years 1915 and 1916, and with the death rates among males of the ages between 20 and 45 in the United States registration area and in the insured whites of the largest insurance company in the United States.

*U. S. Army Enlisted Troops (Monthly Average).*

| | | | |
|---|---|---|---|
| 1905-14. . . . . . | 21.3 | 2.67 | 12.7 |
| 15. . . . . . | 24.8 | 3.08 | 12.6 |
| 16. . . . . . | 21.3 | 3.00 | 14.2 |

*Civil Population in United States (Monthly Average).*

| Registration Area Males. | No. of cases of Pneumonia per 100,000 per month. | No. of deaths from Pneumonia per 100,000 per month. | No. of deaths from Pneumonia per 100 cases of Pneumonia. |
|---|---|---|---|
| Age 20-24. . . . . | — | 3.03 | — |
| 25-34. . . . . | — | 4.24 | — |
| 35-44. . . . . | — | 7.70 | — |
| Met. Life Ins. Co. Insured White Males. | | | |
| Age 20-24. . . . . | — | 2.65 | — |
| 25-34. . . . . | | 5.78 | — |
| 35-44. . . . . | — | 12.08 | — |

The contributing causes of the high diseases incidence above detailed are insufficient warm clothing and blankets, lack of facilities for drying of clothing, in some instances fatigue from long drilling or labor, and insufficient floor space per capita to permit of sanitary sleeping conditions.

The pre-War standard in the U. S. Army for barrack floor and air space was 60 sq. ft. and approximately 600 cu. ft. per capita. Owing to the restrictions put upon materials and transportation, the British have established a minimum standard of 40 sq. ft. of floor space per capita and approximately 400 cu. ft. air space per capita. The regulations governing floor space and cubic air space allowed to French soldiers in barracks provide for a minimum of 32.8 sq. ft. and 529 cu. ft. respectively so far as possible. The limitations placed upon accommodation in the A. E. F. allow but 20 sq. ft. of floor space and 200 cu. ft. of air space per capita.

There are a few other special groups of diseases which are worthy of note :

| | |
|---|---|
| Colitis<br>Entero-Colitis<br>Diarrhea<br>Gastritis<br>Constipation | 42,012 days lost |

These may be properly attributed to limited, unsuitable food and drink, or errors in its preservation, transportation, preparation, or serving.

A loss of 10,700 days from Hypertrophied tonsils and peritonsillar abscesses could have been prevented by suitable operative removal in childhood.

Proper training in posture and walking, and well fitting shoes would have prevented most of the loss of 10,747 from flat foot.

If company discipline, adequate supply of dry clean socks, and shoes and daily attention to the toilet of the feet in winter had prevailed there would not have been the loss of 5,277 days from trench foot and chilblains.

Similarly, if so many men had not been obliged to work in wet clothes and sleep in them too, in winter weather with insufficient covering at night, we might have expected less rheumatic fever and acute synovitis and arthritis which together caused a loss of 54,970 days.

More thorough examination and suitable treatment surgically before joining the A. E. F. would have avoided a loss of 24,848 days from hernia.

Complications of venereal disease, or results of them, are found in phimosis and orchitis which added 10,575 to the days lost.

From the three disease groups :

Abscess of the Skin,
Furunculosis & Pyodemias,
Scabies, there was a total loss of 25,563 days. A loss largely avoidable by the thorough application of well-known and well developed methods of prevention and treatment, as was shown recently by a division in the front line which reduced its Scabies from over 400 to 4 cases in one month with corresponding reduction of skin infections.

A total of 6,780 days lost from lymphadenitis has a fairly direct relationship with the losses from skin infections.

That there were so few losses from diseases of the mouth, stomatitis and gingivitis, speaks well for the dental and oral hygiene; 2,584 days.

Although a total of 28,503 days [from tuberculosis of all kinds is properly classed as among preventable communicable diseases of civil life, there is no reason to believe there was any new infection or spread of infection to others among the 750 tuberculosis patients (pulmonary, bone, etc.) who have been under treatment in the A. E. F.

We may be excused for expressing gratification in the small loss from mental diseases which amounts to 9,773 days.

Valvular heart diseases, other diseases of heart nephritis, acute and chronic, which come so high on the list of causes of death in civilian populations, show a relatively low incidence even for the age group with which we are dealing; loss of 23,755 days.

Alcoholism is responsible for only 1,235 days lost.

The most serious warning which these figures give is that the penalty we must pay for overcrowding of barracks and billets and inadequate provision of facilities for personal cleanliness, provision of warm dry clothing, is an unnecessarily high case rate of the diseases known to be transmitted by personal contact through the discharges of the upper respiratory tract. This is our most urgent problem at the moment.

Roughly, the prevention of much of the avoidable disease here reported falls under the following six headings :

1. Such education and restraint as will prevent the transmission of inherited feeble mindedness and insanity through marriage of the unfit, and such care of the growing child as will bring it to maturity in good health.

2. Elimination of the physically and mentally unfit at the time of application or drafting for enlistment.

Neither of these resources is within the range of the functions of the Medical Department of the A. E. F.

3. Protecting susceptible individuals by artificial immunization, smallpox, typhoid, diphtheria, etc., i. e., doing things to the men by authority under Army regulations.

4. Protecting foods and fluids at source, in transit, storage, preparation and serving, e. g., testing preserved foods, and chlorinating water, i. e., doing things for the men by authority.

5. Maintaining discipline, orderliness, cleanliness, and standards of living, e. g., policing camps, providing prophylactic stations, etc.

6. Teaching by example, by written and spoken word, and by all the various appeals through educational methods, the principles and practice of personal hygiene.

